# Water Conveniences

**Published:** 1866-03

**Authors:** 


### WATER CONVENIENCES.

If water is not supplied by artificial means, so as to come into the kitchen by pipes and a faucet, it should be arranged to have the well or cistern or spring deliver its supply in an apartment immediately adjoining the kitchen, on the same level, and without going outside the house. It can not be truthfully denied that multitudes of women lose health and life itself every year by having to step out from the dry, warm floor of the kitchen upon the cold stones and wet path outside, going to the spring, the wood-yard, or the "smoke-house." And with the experiences and harrowing narrations which daily come to physicians from this direction, that farmer is criminally remiss who, in building a new house, or reconstructing an old one, does not arrange to have a dry and level floor for those who do the cooking, washing, and general housework of the family; so as to make dairy, cellar, wood-house, water-closets, and smoke-house easily accessible by a dry pathway.

